# “Lantern-Shaped” Platinum(III) Complexes with Axially Bound 9-Ethylguanine or 1-Methylcytosine (L) of General Formula [Pt_2_{HN=C(Bu^t^)O}_4_L_2_](NO_3_)_2_

**DOI:** 10.1155/2010/102863

**Published:** 2010-06-08

**Authors:** Concetta Pacifico, Francesco Paolo Intini, Fiorentin Nushi, Giovanni Natile

**Affiliations:** Dipartimento Farmaco-Chimico, Università degli Studi di Bari, Via E. Orabona 4, 70125 Bari, Italy

## Abstract

The synthesis, NMR characterization, and X-ray crystallography of “lantern-shaped” platinum(III) complexes with four pivaloamidate bridging ligands and two 9-ethylguanines (9-EtG) or 1-methylcytosines (1-MeC) in axial positions are reported: *cis-*N^2^O^2^-[Pt_2_{HN=C(Bu^t^)O}_4_(9-EtG)_2_](NO_3_)_2_ and *cis*-N^2^O^2^-[Pt_2_{HN=C(Bu^t^)O}_4_(1-MeC)_2_](NO_3_)_2_. The last complex is, to the best of our knowledge, the first dinuclear compound of platinum(III) with axially bound 1-MeC.

## 1. Introduction

The interest in dinuclear platinum(III) complexes is steadily increasing because of their very interesting chemical properties. They contain a metal-metal single bond which is generally supported by two or four bridging ligands (the latter generally indicated as “lantern shaped” complexes) ([1–3] and references therein). Only few exceptions with three bridging ligands [4], or unsupported by covalent bridges [5, 6], have been so far reported. Usually the bridging ligands form five-member rings comprising the two platinum centers and a set of three atoms providing a suitable bite, for example, NCO [6–23] (including pyrimidine nucleobases), NCS, NCN, SCS, OXO (X = C, S, P), or PXP (X = O, C) [24–29]. Some of these dinuclear platinum(III) complexes have antitumor activity [30–32] or have shown to act as catalysts for the oxidation of olefins [2, 33, 34]. Dinuclear platinum(III) complexes have equatorial and axial ligands [35]; these latter are invariably more weakly bound, due to the strong *trans* labilizing influence exerted by the intermetallic bond [5, 22]. In previous works we have reported the synthesis and structural characterization of “lantern-shaped” platinum(III) complexes with acetamidate and pivaloamidate (HN=C(R)O^−^, R = Me or Bu^t^) bridging ligands and chloride, phosphine or water axial ligands [22, 23]. We have now extended the investigation to the case of axial ligands being purine and pyrimidine nucleobases. In this paper we report the synthesis and NMR characterization of pivaloamidate “lantern-shaped” platinum(III) complexes with 9-ethylguanine (9-EtG) and 1-methylcytosine (1-MeC). The two complexes, *cis-*N^2^O^2^-[Pt_2_{HN=C(Bu^t^)O}_4_(9-EtG)_2_](NO_3_)_2_ and *cis*-N^2^O^2^-[Pt_2_{HN=C(Bu^t^)O}_4_(1-MeC)_2_](NO_3_)_2_, have also been characterized by X-ray crystallography.

## 2. Experimental

**Physical Measurements**

Elemental analyses were obtained with an Elemental Analyzer mod. 1106 Carlo Erba instrument. ^1^H, ^13^C, and ^195^Pt NMR spectra were recorded with a DPX 300 Avance Bruker instrument. ^1^H and ^13^C chemical shifts are referenced to TMS and ^195^Pt chemical shifts to K_2_PtCl_4_ (1 M in water, *δ* = −1614 ppm).

### 2.1. Synthesis

#### 2.1.1. Starting Materials

Reagent grade chemicals were used as received. *cis*-N^2^O^2^-[Pt_2_{HN=C(Bu^t^)O}_4_(NO_3_)_2_] was prepared as already described in a previous work [23].

#### 2.1.2. cis-N^2^O^2^-[Pt_2_{HN=C(Bu^t^)O}_4_(9-EtG)_2_](NO_3_)_2_ **(1)**

*cis*-N^2^O^2^-[Pt_2_{HN=C(Bu^t^)O}_4_(NO_3_)_2_] (50.4 mg, 0.055 mmol) and 9-EtG (20.0 mg, 0.11 mmol) were dissolved in methanol (40 mL) and the reaction solution stirred at 25  °C for 6 hours. The solution was then taken to dryness under reduced pressure and the obtained solid was triturated with chloroform in order to remove unreacted reagents. The suspension was centrifuged and the solid was separated from the solution and dried in a stream of dry air. *Anal.* calc. for C_34_H_58_N_16_O_12_Pt_2_·CHCl_3_·CH_3_OH: C, 30.35; H, 4.46; N, 15.73. Found: C, 30.87; H, 4.60; N, 16.18. ^1^H-NMR (CD_3_OD, ppm): 8.20 (s, H8), 4.31 (q, CH_2_), 1.52 (t, CH_3_), and 1.22 (s, Bu^t^). The compound was obtained in crystalline form (green crystals) from a methanol solution layered under tetrahydrofurane (THF). 

#### 2.1.3. cis-N^2^O^2^-[Pt_2_{HN=C(Bu^t^)O}_4_(1-MeC)_2_](NO_3_)_2_ **(2)**

*cis*-N^2^O^2^-[Pt_2_{HN=C(Bu^t^)O}_4_(NO_3_)_2_] (129.0 mg, 0.14 mmol) and 1-MeC (35.3 mg, 0.28 mmol) were dissolved in methanol (30 mL). The reaction mixture was stirred at 25  °C for 6 hours. The green solution was taken to dryness under reduced pressure and the solid dried in a stream of dry air. *Anal.* calc. for C_30_H_54_N_12_O_12_Pt_2_·H_2_O·CH_3_OH: C, 30.64; H, 4.98; N, 13.83. Found: C, 30.64; H, 4.65; N, 13.81. ^1^H-NMR (CD_3_OD, ppm): 7.83 (d, H6), 5.95 (d, H5), 3.45 (s, CH_3_), and 1.23 (s, Bu^t^). The compound was obtained in crystalline form (yellow crystals) from an ethanol solution layered under 1,4-dioxane. 

### 2.2. X-Ray Crystallography

Selected crystals of compounds **1** and **2** were mounted on a Bruker AXS X8 APEX CCD system equipped with a four-circle Kappa goniometer and a 4K CCD detector (radiation MoK*α*). For data reduction and unit cell refinement the SAINT-IRIX package was employed [36].

For compound **1, **that crystallizes from CH_3_OH/tetrahydrofurane incorporating a molecule of tetrahydrofurane per molecule of compound (**1**·C_4_H_8_O), a total of 42660 reflections (Θ_max _ = 25.18°) were collected. For compound **2**, that crystallizes from CH_3_CH_2_OH/1,4-dioxane incorporating two molecules of 1,4-dioxane per molecule of compound (**2**·2C_4_H_8_O_2_), a total of 43490 reflections (Θ_max _ = 34.11°) were collected. All reflections were indexed, integrated, and corrected for Lorentz, polarization, and absorption effects using the program SADABS [37]. 

The unit cell dimensions were calculated from all reflections and the structures were solved using direct methods technique in the *P *2_1_/*c *space group. 

The model was refined by full-matrix least-square methods. All non-hydrogen atoms were refined anisotropically, except for atoms of *tert*-butyl group (disordered in the case of **2**) and of solvent of crystallization (disordered tetrahydrofurane for **1** and disordered 1,4-dioxane for **2**) that required isotropic treatment in order to maintain satisfactory thermal displacement parameters. 

In the case of complex **1**, the hydrogen atoms were located by Fourier difference and refined isotropically except for the hydrogen atoms of the *tert*-butyl groups that were placed at calculated positions and refined given isotropic parameters equal to 1.5 times the *U*(eq) of the atom to which they are bound. 

In the case of complex **2**, all hydrogen atoms were placed at calculated positions and refined given isotropic parameters equivalent to 1.5 (methyl groups) or 1.2 (other groups) times those of the atom to which they are attached.

All calculations and molecular graphics were carried out using SIR2002 [38], SHELXL97 [39], PARST97 [40, 41], WinGX [42], and ORTEP-3 for Windows packages [43]. Details of the crystal data are listed in Table 1. Selected bond lengths and angles are listed in Table 2.

CCDC-762181 (**1**) and CCDC-762182 (**2**) are available. These data can be obtained free of charge via http://www.ccdc.cam.ac.uk/conts/retrieving.html, or from the Cambridge Crystallographic Data Centre, 12 Union Road, Cambridge CB2 1EZ, UK; fax: (+44) 1223-336-033; or e-mail: deposit@ccdc.cam.ac.uk.

## 3. Results and Discussion

### 3.1. Synthesis and Characterization

9-EtG and 1-MeC both react with lantern shaped [Pt_2_{HN=C(Bu^t^)O}_4_(NO_3_)_2_] (which has a *cis*-N^2^O^2^  configuration on both platinum subunits), in methanol, giving, respectively, compounds **1** and **2** in almost quantitative yields. The new formed complexes exhibit single ^195^Pt NMR signals (−69.8 and 28.2 ppm for **1** and **2**, respectively, solvent CD_3_OD + 10% H_2_O), which are indicative of dinuclear Pt(III) species with symmetrical capping of the axial sites (the precursor complex, [Pt_2_{HN=C(Bu^t^)O}_4_(NO_3_)_2_], resonates at −4.41 ppm in CD_3_OD). The ^1^H-NMR spectrum in CD_3_OD + 10% H_2_O of complex **1** exhibits a single set of signals for 9-EtG with frequencies at 11.32, 8.17, 6.71, 4.29, and 1.51 ppm assigned, respectively, to NH, H(8), NH_2_, CH_2_, and CH_3_ protons (corresponding signals of free 9-EtG fall at 10.85, 7.78, 6.30, 4.07, and 1.40 ppm, respectively). The 0.40 ppm downfield shift of the 9-EtG H8 proton suggests that the coordination occurs through N7. One set of signals is also observed for the pivaloamidate ligands with resonance peaks at 8.66 and 1.23 ppm assigned, respectively, to NH and *tert*-butyl protons (the corresponding protons in the precursor complex [Pt_2_{HN=C(Bu^t^)O}_4_(NO_3_)_2_] resonate at 7.54 and 1.22 ppm, respectively). The deshielding of about 1 ppm observed for the amidic protons of the pivaloamidate ligands may be attributed to the interaction with the guanine base in apical position (see following discussion). 

The ^1^H-NMR spectrum of compound **2 **in CD_3_OD + 10% H_2_O exhibits one set of signals for 1-MeC with resonance peaks at 8.82 and 6.82  (these first two peaks exhibiting a strong exchange peak in the 2D NOESY experiment), 7.83, 5.95, and 3.45 ppm assigned, respectively, to the two unequivalent aminic protons and to H(6), H(5), and methyl group (corresponding signals of free 1-MeC fall at 7.18 (broad singlet), 7.55, 5.85, and 3.35 ppm). The unequivalence of the aminic protons in coordinated 1-MeC is due to the partial double bond character of the C4–N4 linkage, which is reinforced by the metal coordination to N3 [44, 45]. The average deshielding of the aminic protons of 1-MeC, as a consequence of coordination to platinum, is 0.64 ppm. However, while one proton, presumably that pointing towards platinum, undergoes a very large deshielding (1.64 ppm), the other proton undergoes a slight upfield shift (0.36 ppm). The pivaloamidate ligands exhibit one signal for the iminic proton at 8.05 ppm and one signal for the *tert*-butyl groups at 1.23 ppm. The cross peak between the signals at 8.05 and 1.23 ppm, observed in the 2D NOESY spectrum, supports this assignment. Coordination of 1-MeC in apical position causes a deshielding of the amidic protons of the pivaloamidate ligands which is less than half that observed for coordination of 9-EtG (0.51 as compared to 1.12 ppm).

### 3.2. X-Ray Diffraction Analysis

#### 3.2.1. [Pt_2_{HN=C(*B* *u* ^*t*^)O}_4_(9-EtG)_2_](NO_3_)_2_ **(1)**

**Figure 1:**
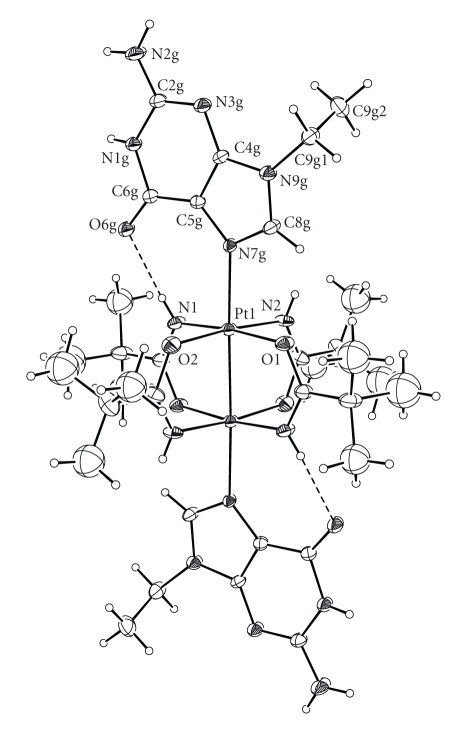
View of the [Pt_2_{HN=C(Bu^t^)O}_4_(9-EtG)_2_]^2+^ complex showing the atomic numbering scheme of most important atoms. Displacement ellipsoids are drawn at 20% probability level.

Complex **1 **crystallizes incorporating one molecule of THF per molecule of complex. The asymmetric unit comprises half molecule of complex and half molecule of THF and the structure is generated by inversion at the midpoint of the Pt–Pt bond (Figure 1). Each platinum(III) atom has distorted octahedral geometry with the N7 of 9-EtG and the second platinum subunit in axial positions.

The Pt–Pt distance (2.4512(5) Å) is closer to that of the analogous complex with axial chlorides ([Pt_2_{HN=C(Bu^t^)O}_4_Cl_2_], 2.448(2) Å) than to that of the complexes with one or two axial triphenylphosphine ligand(s) ([Pt_2_{HN=C(Bu^t^)O}_4_(PPh_3_)(H_2_O)](NO_3_)_2,_ 2.468(1) Å; [Pt_2_{HN=C(Bu^t^)O}_4_(PPh_3_)_2_](NO_3_)_2_, 2.504(1) Å) [22, 23]. Thus the Pt–Pt distance is influenced by the nature of the axial ligands and an N7-coordinated guanine appears to exert a *trans* influence similar to that of a chloride. The platinum coordination squares are perfectly eclipsed (maximum twist angle 1.5°); such a conformation allows the greatest separation between the platinum atoms. The platinum atoms are displaced from the equatorial coordination planes by 0.087 Å towards the axial 9-EtG, such a displacement being a measure of the strength with which the four bridging ligands pull together the two metal centers.

The equatorial Pt–N [1.993(7)–1.996(6) Å] and Pt–O distances [2.019(5)–2.035(5) Å] are in the range of those reported for doubly and quadruply bridged dinuclear platinum(III) [17, 22, 23, 46], four-coordinate platinum(II), and six-coordinate platinum(IV) complexes [4].

As expected, the axial Pt(III)–N7 bond length (2.200(5) Å in **1**) is longer than those typically seen in 4-coordinate platinum(II) and 6-coordinate platinum(IV) guanine complexes (~1.96–2.11 Å) [47–51]. The lengthening can be ascribed to the strong *trans* influence exerted by the Pt–Pt bond. We also notice that the Pt(III)–N7 bond is slightly longer in **1** than in analogous dinuclear Pt(III) species (e.g., 2.189(6) Å in *ht*-*cis*-[Pt_2_(NH_3_)_4_(1-Mec-N3,N4)_2_(9-EtG)_2_](ClO_4_)_4_·5H_2_O (*ht* indicates the head-to-tail arrangement of the two bridging 1-MeC ligands) [52], 2.187(6) and 2.181(7) Å in *ht*-*cis*-[Pt_2_(NH_3_)_4_(1-Mec- N3,N4)_2_(9-EtG)_2_](NO_3_)_4_·9H_2_O) [53]. We believe that the longer Pt(III)–N7 bond observed in **1** can be ascribed to a stronger *trans* influence exerted by the Pt–Pt bond, that in **1** is shorter (2.4511(4) Å) than in the latter two complexes (2.587(1) and 2.586(1) Å in *ht*-*cis*-[Pt_2_(NH_3_)_2_(1-Mec-N3,N4)_2_(9-EtG)_2_](ClO_4_)_4_·5H_2_O [52] and in *ht*-*cis*-[Pt_2_(NH_3_)_4_(1-Mec-N3,N4)_2_(9-EtG)_2_](NO_3_)_4_·9H_2_O, respectively) [53].

The guanine is nearly coplanar with a pivaloamidate (C5g–N7g–Pt–N1 torsion angle of 16.4(7)°); this allows the formation of a strong hydrogen bond between the NH of the amidate ligand and the O6 of guanine (N1 ⋯ O6g = 2.80(1) Å, (N1)H1 ⋯ O6g = 2.07(9) Å, N1–H1 ⋯ O6g = 166(9)°). The resulting seven-member ring motif can be defined as S(7) by Etter's graph-set notation [54]. The orientation of the guanine in **1** is very similar to that found in the tetrabridged dirhodium(II) complex [Rh_2_{O=C(CH_3_)O}_2_{HN=C(CF_3_)O}_2_(9-EtG)_2_] [55] also containing a strong H-bond (N ⋯ O6g = 2.94(2) Å, (N1)H1 ⋯ O6g = 2.20(9) Å, N1–H1 ⋯ O6g = 158(1)°). It appears that a pivaloamidato ligand is as good as the trifluoroacetamidato ligand in forming such an H-bond.

**Figure 2:**
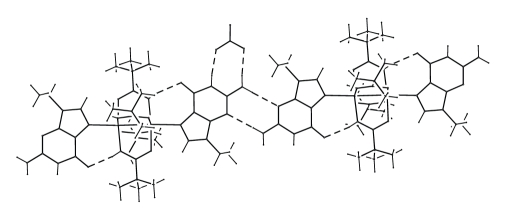
View of the H-bonds along the chains of [Pt_2_{HN=C(Bu^t^)O}_4_(9-EtG)_2_]^2+^ complexes.

The crystal packing is mainly governed by two symmetrical hydrogen bonds involving N2 and N3 of two adjacent guanines (N2g ⋯ N3g^ii^ = 3.07(1) Å, (N2g)H21g ⋯ N3g^ii^ = 2.22(1) Å, N2g–H21g ⋯ N3g^ii^ = 175(1)°; *i* *i* = − *x* + 1, −*y* + 1, −*z* + 1), forming a centrosymmetric eight-member ring. This ring motif can be defined as R_2_^2^(8) by Etter's graph-set notation (Figure 2). These H-bonds allow the formation of chains of complexes extending, alternatively, parallel to the (110) and to the ($1\bar{1}0$) directions. The angle between adjacent chains is 66°.

Different chains are linked by nitrate anions. The nitrate anion is anchored to the guanine base through two strong H-bonds (N1g ⋯ O5 = 2.81(1) Å, (N1g)H1g ⋯ O5 = 1.99(8) Å, N1g–H1g ⋯ O5 = 173(7)°; N2g ⋯ O4 = 2.87(1) Å, (N2g)H22g ⋯ O4 = 2.02(8) Å, N2g–H22g ⋯ O4 = 171(1)°) forming an eight-member ring (R_2_^2^(8) according to Etter's graph-set notation). The same nitrate anion forms a third hydrogen bond with the amidic NH of an adjacent complex (N2^i^ ⋯ O3 = 3.18(1) Å, (N2)^i^H2^i^ ⋯ O3 = 2.49(8) Å, N2^i^–H2^i^ ⋯ O3 = 157(8)°; *i* = *x*, −*y* + 1/2, *z* − 1/2) with an Etter's graph-set motif of type D.

The THF solvent molecule is disordered, the oxygen atom of 50% of the molecules pointing in the direction opposite to that of the other 50% molecules. As a consequence THF appears as a flat 1,4-dioxane-type molecule with the two oxygens having occupancy factor 0.5 and the carbons occupancy factor 1. The accuracy of the X-ray data is not allowed to distinguish between carbon atoms belonging to the differently oriented THF molecules.

#### 3.2.2. [Pt_2_{HN=C(*B* *u* ^*t*^)O}_4_(1-MeC)_2_](NO_3_)_2_ **(2)**

**Figure 3:**
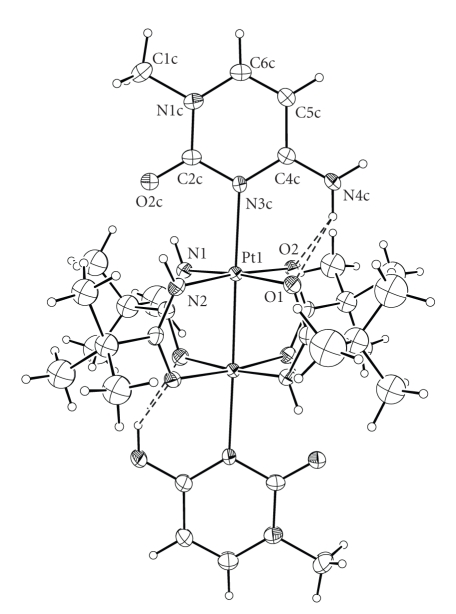
View of the [Pt_2_{HN=C(Bu^t^)O}_4_(1-MeC)_2_]^2+^ complex showing the atomic numbering scheme of most important atoms. The *tert*-butyl groups are disordered and can occupy two different positions; only one position is shown for clarity. Displacement ellipsoids are drawn at 20% probability level.

Compound **2 **crystallizes incorporating two molecules of 1,4-dioxane per molecule of complex. The asymmetric unit comprises half molecule of complex and one of dioxane and the structure is generated by inversion at the midpoint of the Pt–Pt linkage (Figure 3). Each Pt(III) atom has a distorted octahedral geometry with the N3 of 1-MeC and the second platinum subunit in axial positions.

The Pt–Pt distance (2.4523(4) Å) is very similar to the analogous distance observed in compound **1**. As for **1**, the platinum coordination squares are perfectly eclipsed (maximum twist angle of 0.8°), and the platinum atoms are displaced from the equatorial coordination planes by 0.090 Å towards the axial cytosine. Also the equatorial Pt–N [1.967(5) and 1.982(5) Å] and Pt–O distances [2.011(4) and 2.025(4) Å] are in the range of those observed in **1** and reported for four-coordinate platinum(II) and six-coordinate platinum(IV) complexes [4].

**Figure 4:**
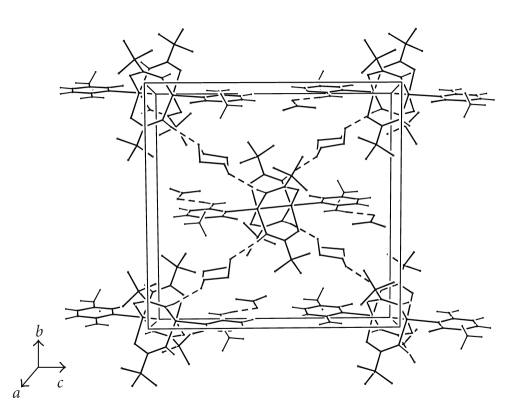
View of the crystal packing along the *a* direction for [Pt_2_{HN=C(Bu^t^)O}_4_(1-MeC)_2_](NO_3_)_2_ *·*2(1,4-dioxane). Since the *tert*-butyl groups and the dioxane molecules are disordered, only one occupation site is shown for clarity.

As expected, the axial Pt(III)–N3c bond length (2.230(5) Å) is longer than that observed in 4-coordinate platinum(II) and 6-coordinate platinum(IV) complexes with cytosine (~2.025–2.082 Å) [45, 47, 56–58]. It is to be noted that the Pt–N3c distance is slightly longer than the Pt–N7g distance observed in compound **1**. The cytosine plane bisects the angle between two adjacent pivaloamidate planes forming a C2c–N3c–Pt–N1 torsion angle of 44°. This allows the formation of bifurcated hydrogen bonds between N4 of 1-MeC and the O atoms of two amidate ligands (N4c ⋯ O1 = 2.92(1) Å, (N4c)H41c ⋯ O1 = 2.30(1) Å, N4c–H41c ⋯ O1 = 130(1)°; N4c ⋯ O2 = 2.93(1) Å, (N4c)H41c ⋯ O2 = 2.31(1) Å, N4c–H41c ⋯ O2 = 130(1)°) [59]. Each nitrate anion (nitrate oxygens O6, O7, and O8) is anchored to one 1-MeC through a hydrogen bond (N4c ⋯ O8 = 2.85(1) Å, (N4c)H42c ⋯ O8 = 2.00(1) Å, N4c–H42c ⋯ O8 = 167(1)°, Figure 4).

In the crystal packing, complex molecules are located (with the inversion center) at the four corners and at the center of face A. The crystal packing is mainly governed by hydrogen bonds between complexes and 1,4-dioxane molecules. Each molecule of 1,4-dioxane (1,4-dioxane oxygens O4 and O5) bridges two adjacent molecules of complex (N2 ⋯ O4 = 2.98(1) Å, (N2)H2 ⋯ O4 = 2.19(1) Å, N2–H2 ⋯ O4 = 152(1)°; N1^i^ ⋯ O5 = 3.02(1) Å (N1^i^)H1^i^ ⋯ O5 = 2.25(1) Å, N1^i^–H1^i^ ⋯ O5 = 149(1)°; *i* = − *x*, −*y* + 1/2, *z* − 1/2). In this way each molecule of complex is surrounded by four molecules of dioxane connecting the former complex to the four adjacent complex molecules on face A (Figure 4).

The *tert*-butyl groups are disordered and each set of three methyl groups can occupy two different positions, each position with occupancy factor 0.5. Also the 1,4-dioxane solvent molecules are disordered. The position is fixed for the two oxygen atoms while the four carbon atoms can occupy two different positions each one with occupancy factor 0.5. In each case the 1,4-dioxane molecule adopts a chair conformation.

## 4. Conclusions

The coordination of 9-EtG and 1-MeC to the axial sites of quadruply bridged dinuclear species of platinum(III) has been established. The complexes are stable in solution as well as in the solid state. Complex **1 **is one of the few examples of dinuclear platinum(III) species with axially bound guanines, while complex **2 **is, to the best of our knowledge, the first compound of this type (axially bound 1-MeC). The axial Pt–N3 bond in **2** is 0.010 Å longer than the axial Pt–N7 bond in **1**. Since the Pt–Pt core is very similar in the two cases, we argue that the longer distance found in **2** is indicative of a weaker binding of 1-MeC as compared to 9-EtG. Previous attempts to bind 1-MeC in the axial positions of a dinuclear platinum(III) complex, (e.g., *cis*-[Pt_2_(NH_3_)_4_(1-Mec- N3,N4)_2_ *X* *Y*]*Z*_*n*_, *X* and *Y* stand for axial ligands of different types and *Z* stands for counteranion(s)) have been unsuccessful [52]. In contrast our dinuclear Pt(III) core, with four pivaloamidate bridging ligands, readily binds nucleobases, comprising 1-MeC, forming stable compounds. It is possible that the presence in the equatorial platinum coordination plane of groups with good H-bond donor/acceptor properties, and therefore able to establish additional bonds with the apical ligands, gives a decisive contribution to the formation of such complexes. H-bond interaction causes, in the case of **1**, a downfield shift of the pivaloamidate amidic proton by 1.12 ppm and, in the case **2,** a downfield shift of one aminic proton of 1-MeC by 1.64 ppm. In the latter case the H-bond is bifurcated and the 1-MeC aminic proton, (N4c)H41c, interacts, simultaneously, with the oxygen atoms of two *cis* pivaloamidate ligands. In principle, the 1-MeC could form, in addition to the H-bond described above, also an H-bond between the 1-MeC oxygen, O2c, and the pivaloamidate amidic protons; such an H-bond, however, does not form or is extremely weak (downfield shift of the amidic proton of only 0.51 ppm as compared to 1.12 ppm observed in compound **1**). A possible cause of weakness of the latter H-bond is the dihedral angle of 45° between 1-MeC and pivaloamidate planes; such an angle is optimal for the bifurcated H-bond involving the aminic group but is detrimental for a potential H-bond involving the 1-MeC oxygen. In fact it appears that, while a proton can interact with two oxygens (bifurcated H-bond), one oxygen can only interact with one proton (regular H-bond as observed in compound **1**).

“Lantern shaped” platinum(III) complexes have been shown, by Cervantesand coworkers, to be endowed with antitumor activity (e.g., [Pt_2_(2-mercaptopyrimidine)_4_Cl_2_] and [Pt_2_(2-mercaptopyridine)_4_Cl_2_]) [30–32]. It will be worth investigating the antitumor activity of our amidate complexes for which we have shown a greater propensity to form adducts with nucleobases in apical positions.

## Figures and Tables

**Table 1:** Crystal data and structure refinement parameters for [Pt_2_{HN=C(Bu^t^)O}_4_(9-EtG)_2_](NO_3_)_2_·tetrahydrofurane (**1**·C_4_H_8_O) and [Pt_2_{HN=C(Bu^t^)O}_4_(1-MeC)_2_](NO_3_)_2_·2(1,4-dioxane) (**2**·2C_4_H_8_O_2_).

Crystal	**1**·C_4_H_8_O	**2**·2C_4_H_8_O_2_
Empirical formula	C_38_H_66_N_16_O_13_Pt_2_	C_38_H_70_N_12_O_16_Pt_2_
Formula weight	1345.24	1341.24
Temperature (K)	293(2)	293(2)
Wavelength (Å)	0.71073	0.71073
Crystal system	monoclinic	monoclinic
Space group	*P *2_1_/*c *	*P *2_1_/*c *
*a* (Å)	9.7776(3)	10.6566(5)
*b* (Å)	14.8367(5)	15.8137(6)
*c* (Å)	18.8734(7)	15.5868(6)
*β* *(*°)	98.54(1)	102.51(1)
Volume (Å^3^)	2707.5(2)	2564.5(3)
*Z*	2	2
Density (calculated) (Mg/m^3^)	1.650	1.737
Absorption coefficient (mm^−1^)	5.231	5.524
*F*(000)	1332	1332
Crystal size (mm^3^)	0.300 × 0.150 × 0.080	0.240 × 0.210 × 0.075
*θ* range for data collection (°)	1.75 to 25.18	2.34 to 34.11
Index ranges	−11 ≤ *h* ≤ 11, −17 ≤ *k* ≤ 17, −22 ≤ *l* ≤ 22	−16 ≤ *h* ≤ 16, −24 ≤ *k* ≤ 24, −23 ≤ *l* ≤ 23
Reflections collected	42660	43490
Independent reflections	4843 [*R*(int ) = 0.0917]	10083 [*R*(int ) = 0.0639]
Refinement method	Full-matrix least-squares on *F* ^2^	Full-matrix least-squares on *F* ^2^
Data/restraints/parameters	4843/0/301	10083/0/291
Goodness-of-fit on *F* ^2^	1.046	1.003
Final *R* indices [*I* > 2*σ*(*I*)]	*R*1 = 0.0398, *w* *R*2 = 0.0906	*R*1 = 0.0469, *w* *R*2 = 0.1117
*R* indices (all data)	*R*1 = 0.0614, *w* *R*2 = 0.1014	*R*1 = 0.0968, *w* *R*2 = 0.1390
Largest diff. peak and hole (*e* Å^−3^)	1.372 and −0.751	2.571 and −0.766

**Table 2:** Bond lengths [Å] and angles [°] for [Pt_2_{HN=C(Bu^t^)O}_4_(9-EtG)_2_](NO_3_)_2_·tetrahydrofurane (**1**·C_4_H_8_O) and [Pt_2_{HN=C(Bu^t^)O}_4_(1-MeC)_2_](NO_3_)_2_·2(1,4-dioxane) (**2**·2C_4_H_8_O_2_).

Crystal	**1**·C_4_H_8_O	**2**·2C_4_H_8_O_2_
Pt–N1	1.993(7)	1.967(5)
Pt–N2	1.996(6)	1.982(5)
Pt–O2	2.019(5)	2.025(4)
Pt–O1	2.035(5)	2.011(4)
Pt–Pt^a,b^	2.4512(5)	2.4523(4)
Pt–N7g	2.200(5)	
Pt–N3c		2.230(5)
		
N1–Pt–N2	90.6(3)	92.0(2)
N1–Pt–O2	90.4(3)	88.2(2)
N2–Pt–O1	90.4(2)	88.4(2)
O1–Pt–O2	88.1(2)	90.9(2)
N1–Pt–N7g	97.7(2)	
N2–Pt–N7g	94.3(2)	
O1–Pt–N7g	87.4(2)	
O2–Pt–N7g	90.4(2)	
Pt^a^–Pt–N7g	177.2(1)	
N1–Pt–N3c		95.3(2)
N2–Pt–N3c		95.4(2)
O1–Pt–N3c		89.9(2)
O2–Pt–N3c		89.8(2)
Pt^b^–Pt–N3c		179.0(1)

Symmetry transformations used to generate equivalent atoms: (a) −*x*, −*y*, −*z* + 1 for crystal **1**·C_4_H_8_O and (b) −*x*, −*y*, −*z* for crystal **2**·2C_4_H_8_O_2_.
